# Laryngocele: a rare complication of surgical tracheostomy

**DOI:** 10.1186/1471-2482-6-14

**Published:** 2006-11-27

**Authors:** Tahwinder Upile, Waseem Jerjes, Fabian Sipaul, Mohammed El Maaytah, Sandeep Singh, David Howard, Colin Hopper, Anthony Wright

**Affiliations:** 1 Oral & Maxillofacial Surgery/Head & Neck Unit, University College London Hospitals, London, UK; 2 The Royal National Throat, Nose and Ear Hospital, London, UK

## Abstract

**Background:**

A laryngocele is usually a cystic dilatation of the laryngeal saccule. The etiology behind its occurrence is still unclear, but congenital and acquired factors have been implicated in its development.

**Case presentation:**

We present a rare case of laryngocele occurring in a 77-year-old Caucasian woman. The patient presented with one month history of altered voice, no other associated symptoms were reported. The medical history of the patient included respiratory failure secondary to childhood polio at the age of ten; the airway management included a surgical tracheostomy.

Flexible naso-laryngoscopy revealed a soft mass arising from the posterior pharyngeal wall obscuring the view of the posterior commissure and vocal folds. The shape of the mass altered with respiration and on performing valsalva maneuver. A plain lateral neck radiograph revealed a large air filled sac originating from the laryngeal cartilages and extending along the posterior pharyngeal wall. The patient was then treated by endoscopic laser marsupialization and reviewed annually.

We discuss the complications of tracheostomy and the pathophysiology of laryngoceles and in particular the likely aetiological factors in this case.

**Conclusion:**

A laryngocele presenting in a female patient with tracheostomy is extremely rare and has not been to date reported in the world literature. A local mechanical condition may be the determinant factor in the pathogenesis of the disease.

## Background

A laryngocele is usually a cystic dilatation of the laryngeal saccule. The etiology behind its occurrence is still unclear, but congenital and acquired factors have been implicated in its development [1,2].

Laryngoceles appear to be an atavistic remnant from the higher apes, particularly those who use their arms with the thoracic cage fixed whilst swinging through the trees. In an excellent review of 139 laryngoceles, Stell and Maran showed that the sex incidence is 5:I in favor of men, and the maximum age incidence is in the sixth decade. The authors suggested that two out of three laryngoceles are unilateral and they may be combined, external or internal, with roughly equal frequency; about 8 per cent become infected and present as pyocele. Furthermore they reported as case where the laryngocele could have been caused by prolonged and repeated valsalva. They suggested that the increased air pressure in the larynx may make an already existing laryngocele manifest [1]. In another review of the aetiology of this entity it was suggested that local laryngeal pathologies are perhaps the main determinant factors in the pathogenesis of the disease [2].

Congenitally, the laryngeal saccule is a remnant corresponding to the lateral laryngeal air sacs of the higher anthropoid apes, which may on occasion, manifest suddenly in response to pressure caused by coughing, straining at stool, or trumpet playing (i.e. valsalva maneuvers). An acquired laryngocele may develop when the laryngeal ventricle becomes functionally obstructed as a result of an increase in intraglottic pressure, e.g. excessive coughing, playing a wind instrument or obstruction of appendicular ostium [1,2].

Three types of laryngoceles are described. An internal laryngocele is confined to the interior of the larynx and extends posterosuperiorly into the false vocal cord and the aryepiglottic fold; this type appears on laryngoscopy as a smooth swelling of the supraglottis. An external laryngocele extends superiorly to appear laterally in the neck through the opening in the thyrohyoid membrane for the superior laryngeal nerve and vessels; these clinically present as a swelling in the neck at the level of the hyoid bone anterior to the sternocleidomastoid muscle. The simultaneous existence of both features is termed a combined laryngocele [1-4].

The simple laryngocele is an uncomplicated air-filled dilatation of the appendix of the laryngeal ventricle. When the neck of the laryngocele is obstructed, it becomes filled with mucus, and air-fluid level or fluid filled laryngocele forms. This lesion is named as laryngopyocele if it is infected. Approximately 10% of laryngoceles become infected. A laryngopyocele usually presents as an airway obstruction and/or an infected neck mass. The symptoms of laryngopyocele include hoarseness, dysphagia, dyspnea, a 'full' sensation in the throat. Glottic obstruction varies from minimal to complete. Fever may not exist in all patients [2-5].

Erdogmus et al. reported a laryngocele in a patient with ankylosing spondylitis. It was believed that its development might be a result of increased intra-abdominal pressure, caused by rheumatoid arthritis, with associated increased intralaryngeal pressure [1].

The relationship between laryngoceles and laryngeal carcinoma is still debated. Akbas et al. presented a case of bilateral asymptomatic laryngoceles in a 45-year-old male patient suffering laryngeal squamous cell carcinoma [6]. Another interesting case implicating cancer with laryngocele was reported in a 70-year-old man, who had a laryngeal tumor discovered by otolaryngological examination during admission for suspicion of facial nerve paralysis. Unfortunately the laryngocele wasn't identified and the patient was found dead in bed on the seventh hospital day. An autopsy revealed an internal type laryngocele in the right larynx and infarction of the left dorso-lateral portion of the medulla oblongata. Suffocation resulted from mucous sputum filling his larynx, which had been narrowed by a laryngocele from the right, in an unfortunate association with movement disturbance of the left larynx caused by the infarction of the left dorso-lateral portion of the medulla oblongata, and was considered the cause of death as no cardiovascular-respiratory problems were identified [2].

A patient with a history of psychiatric disorder presented with severe subcutaneous facial, palpebral and cervical emphysema, dysphonia, dysphagia and slight respiratory difficulty. Fiberoptic bronchoscopy revealed upper airway obstruction due to edema in an intact airway. Successive CT scans gave evidence of hyoid fracture and laryngocele, in addition to the corresponding emphysema of the subcutaneous area and pneumomediastinum [7].

We present a case of laryngocele in a 77-year-old Caucasian female following surgical tracheostomy. To the authors' knowledge, and from a review of the literature, this combination has not been previously described.

## Case presentation

A 77-year-old Caucasian female presented to the Head & Neck Unit/University College London Hospital with one month history of altered voice, no other associated symptoms were reported.

The medical history of the patient included respiratory failure secondary to childhood polio at the age of ten; the airway management included performing a surgical tracheostomy. She had been managed with cuirass ventilation for numerous years but was recently maintained on unassisted ventilation using a silver negus tracheostomy tube. There was no other significant medical, family or social history.

Clinical examination showed she has low volume hoarse voice, but was otherwise normal apart from a tracheostomy.

**Figure 1:**
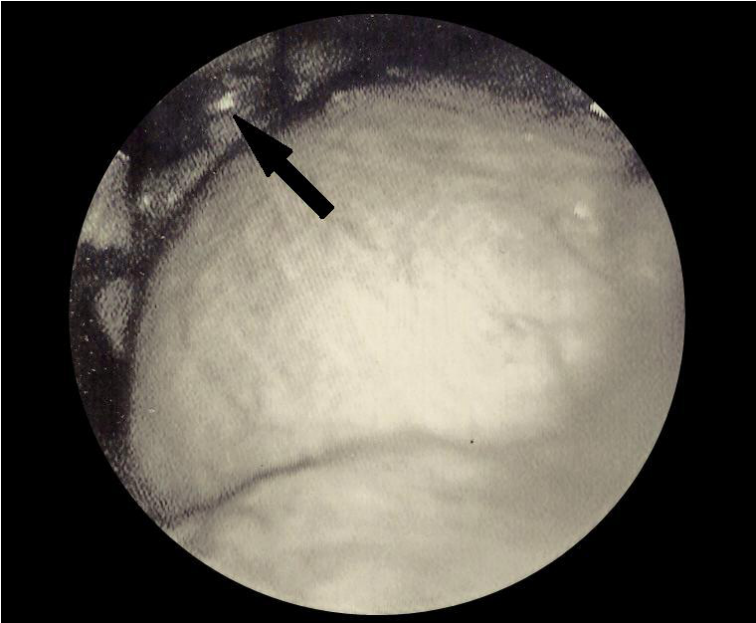
Flexible naso-laryngoscopy showing a soft mass arising from the posterior pharyngeal wall.

**Figure 2:**
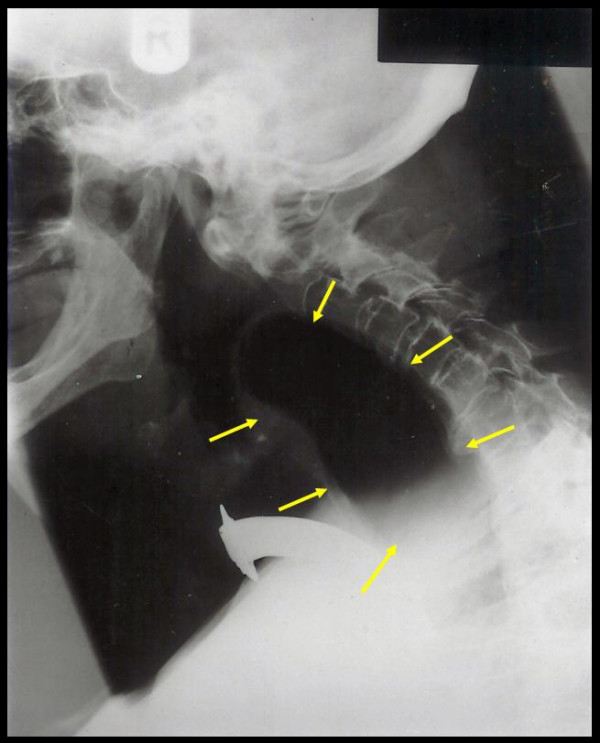
Plain lateral neck radiograph showing a large air filled sac arising from posterior pharyngeal wall.

Flexible naso-laryngoscopy revealed normal but paralyzed vocal folds. Unusually she had a soft mass arising from the posterior pharyngeal wall obscuring the view of the posterior commissure and vocal folds. The shape of the mass altered with respiration and on performing valsalva maneuvers (Figure 1). A plain lateral neck radiograph revealed a large air filled sac originating from the laryngeal cartilages and extending along the posterior pharyngeal wall (Figure 2).

The diagnosis of a laryngocele was primarily made on the basis of the laryngeal examination, confirmed by the presence of an air filled saccular cavity on plain radiography. The patient was then treated by endoscopic laser marsupialization and reviewed annually.

## Conclusion

Complications from surgical procedures are common and must be taken into account when assessing the risks and benefits of a particular treatment approach. Complications following tracheostomy are common and can be early or late (Tables 1 &2); the frequency and severity of occurrence depend on several factors. These include the specific approach to tracheostomy, the skill and experience of the operator, and patient anatomic and physiologic factors. The incidence of undesired outcomes during tracheostomy cannot be exactly predicted because of the interaction of those factors. Treatment modalities vary depending upon the nature of the complication. For the most frequent complication, tracheal stenosis, a multidisciplinary approach utilizing bronchoscopy, laser, airway stents, and tracheal surgery is most effective [8,9].

Our case here is unusual in several respects. Laryngoceles are pathophysiologically thought to arise from abnormal dilatations of the laryngeal ventricle and saccule secondary to pressure changes as a result of an obstruction. However in this patient with a tracheostomy no mechanical rise in pressure should have occurred to cause the development or expansion of the laryngocele. It is possible that trauma at the original tracheostomy placement could have caused an underlying weakness, defect or mechanical obstruction, which may have predisposed to the development of this anomaly exacerbated by cuirass ventilation.

The case is also unusual in that the laryngocele manifested itself as a posterior pharyngeal wall mass whose shape altered with respiration, and whose dimensions increased on valsalva maneuver. It is likely that this was an internal laryngocele which had extended posteriosuperiorly into the ventricular folds, aryepiglottic folds and submucosally into the posterior pharyngeal wall. The swelling was soft on nasoendoscopic palpation.

To date no association has been reported between cuirass ventilation and laryngocele development which is traditionally attributed to localized pressure changes. Hence we feel that the laryngotracheal injury at tracheostomy to be causative. However, one may still convincingly argue that the pathophysiological mechanism of the laryngocele development in this case are still consequent to the action of pressure changes initially acting upon a mucosal weakness through prolonged cuirass ventilation which we feel led to aerocele establishment. Through an understanding of La Place's law and the local histological anatomy of the connective tissues in this region, it is by no stretch of the imagination possible to explain the direction and nature of the expansion of this aerocele after initial traumatic disturbance of the laryngeal epithelium. Further development of the laryngocele can be explained by localized intermittent valsalva pressure build up during phonation with a fenestrated tracheostomy tube and a flutter valve that is in common usage by many long term tracheostomy patients. This would cause raised supraglottic pressure and promote the expansion of the established diverticulum.

The diagnosis is usually made on clinical examination with plain radiography supplemented with further imaging e.g. CT or ideally MRI to assess the origin, connection and exclude other obstructing factors [10,11].

Treatment is primarily surgical, ideally aiming for excision of the saccule at its neck. There is need for careful endoscopic examination with biopsy to exclude the possibility of laryngeal carcinoma in the ventricle as a cause for the laryngocele before definitive surgical procedure. There are many surgical options ranging from an external approach to endoscopic treatment including endoscopic laser marsupialization [10,11].

A laryngocele presenting in a female patient with tracheostomy is extremely rare and has not been to date reported in the world literature. A local mechanical condition may be the determinant factor in the pathogenesis of the disease.

## Competing interests

The author(s) declare that they have no competing interests.

## Authors' contributions

**TU**: designed the study, carried out the literature research, clinical study and manuscript preparation.

**WJ**: carried out the literature research, manuscript preparation, and manuscript review.

**FS**: carried out the manuscript editing and manuscript review.

**ME**: carried out the manuscript editing and manuscript review.

**SS**: carried out the manuscript editing and manuscript review.

**DH**: carried out the manuscript editing and manuscript review.

**CH**: carried out the manuscript editing and manuscript review.

**AW**: designed the study, carried out the literature research, clinical study and manuscript preparation.

All authors read and approved the final manuscript.

## Pre-publication history

The pre-publication history for this paper can be accessed here:



## Figures and Tables

**Table 1:** Complications of Tracheostomy I

Early Complications of Tracheostomy		
Serious	Intermediate	Minor
		
Death	Desaturation/hypotension	Hemorrhage
Arrest	Tracheal wall injury	Difficult tube placement
Pneumothorax	Cannula misplacement	False passage
Pneumomediastinum	Convert to open Aspiration	Subcutaneous emphysema

**Table 2:** Complications of Tracheostomy II

Late Complications of Tracheostomy
Tracheal stenosis (granulation tissue)
Tracheomalacia
Tracheoinnominate-artery fistula
Tracheoesophageal fistula
Pneumonia
Aspiration

## References

[B1] Stell PM, Maran AG (1975). Laryngocoele. J Laryngol Otol.

[B2] Amin M, Maran AG (1988). The aetiology of laryngocoele. Clin Otolaryngol Allied Sci.

[B3] Nazaroglu H, Ozates M, Uyar A, Deger E, Simsek M (2000). Laryngopyocele: signs on computed tomography. Eur J Radiol.

[B4] Rutka J, Birt D (1983). Laryngocele: a case report and review. J Otolaryngol.

[B5] Swartz JD, D'Angelo AJ, Harnsberger HR, Zwillenberg S, Marlowe FI (1990). The laryngeal mucocele. Imaging analysis of a rare lesion. Clin Imaging.

[B6] Akbas Y, Unal M, Pata YS (2004). Asymptomatic bilateral mixed-type laryngocele and laryngeal carcinoma. Eur Arch Otorhinolaryngol.

[B7] Canizares MA, Arnau A, Fortea A, Zarzuela V, Martinez-Vallina P, Canto A (2000). Hyoid fracture and traumatic subcutaneous cervical emphysema from an attempted hanging. Apropos a case. Acta Bronchopneumol.

[B8] Epstein SK (2005). Late complications of tracheostomy. Respir Care.

[B9] Durbin CG (2005). Early complications of tracheostomy. Respir Care.

[B10] Ettema SL, Carothers DG, Hoffman HT (2003). Laryngocele resection by combined external and endoscopic laser approach. Ann Otol Rhinol Laryngol.

[B11] Martinez Devesa P, Ghufoor K, Lloyd S, Howard D (2002). Endoscopic CO2 laser management of laryngocele. Laryngoscope.

